# Framework for developing cost-effectiveness analysis threshold: the case of Egypt

**DOI:** 10.1186/s42506-024-00159-7

**Published:** 2024-06-03

**Authors:** Ahmad N. Fasseeh, Nada Korra, Baher Elezbawy, Amal S. Sedrak, Mary Gamal, Randa Eldessouki, Mariam Eldebeiky, Mohsen George, Ahmed Seyam, Asmaa Abourawash, Ahmed Y. Khalifa, Mayada Shaheen, Sherif Abaza, Zoltán Kaló

**Affiliations:** 1 Faculty of Pharmacy Alexandria University, Alexandria, Egypt; 2 Syreon Middle East, Alexandria, Egypt; 3 Department of Public Health, Cairo University, Cairo, Egypt; 4 Egyptian Authority for Unified Procurement, Medical Supply and Technology Management, Cairo, Egypt; 5 Department of Community Health, Fayoum University, Fayoum, Egypt; 6 Universal Health Insurance Authority, Cairo, Egypt; 7 Egyptian Drug Authority, Cairo, Egypt; 8 World Health Organization Representative Office, Cairo, Egypt; 9 Roche, Cairo, Egypt; 10 Center for Health Technology Assessment, Semmelweis University, Budapest, Hungary; 11 Syreon Research Institute, Budapest, Hungary

**Keywords:** Cost-effectiveness threshold, CET, Multiple thresholds, Egypt, Incremental relative QALY gain, Cost-effectiveness threshold multiplier

## Abstract

**Background:**

Cost-effectiveness analyses rarely offer useful insights to policy decisions unless their results are compared against a benchmark threshold. The cost-effectiveness threshold (CET) represents the maximum acceptable monetary value for achieving a unit of health gain. This study aimed to identify CET values on a global scale, provide an overview of using multiple CETs, and propose a country-specific CET framework specifically tailored for Egypt. The proposed framework aims to consider the globally identified CETs, analyze global trends, and consider the local structure of Egypt’s healthcare system.

**Methods:**

We conducted a literature review to identify CET values, with a particular focus on understanding the basis of differentiation when multiple thresholds are present. CETs of different countries were reviewed from secondary sources. Additionally, we assembled an expert panel to develop a national CET framework in Egypt and propose an initial design. This was followed by a multistakeholder workshop, bringing together representatives of different governmental bodies to vote on the threshold value and finalize the recommended framework.

**Results:**

The average CET, expressed as a percentage of the gross domestic product (GDP) per capita across all countries, was 135%, with a range of 21 to 300%. Interestingly, while the absolute value of CET increased with a country’s income level, the average CET/GDP per capita showed an inverse relationship. Some countries applied multiple thresholds based on disease severity or rarity. In the case of Egypt, the consensus workshop recommended a threshold ranging from one to three times the GDP per capita, taking into account the incremental relative quality-adjusted life years (QALY) gain. For orphan medicines, a CET multiplier between 1.5 and 3.0, based on the disease rarity, was recommended. A two-times multiplier was proposed for the private reimbursement threshold compared to the public threshold.

**Conclusion:**

The CET values in most countries appear to be closely related to the GDP per capita. Higher-income countries tend to use a lower threshold as a percentage of their GDP per capita, contrasted with lower-income countries. In Egypt, experts opted for a multiple CET framework to assess the value of health technologies in terms of reimbursement and pricing.

**Supplementary Information:**

The online version contains supplementary material available at 10.1186/s42506-024-00159-7.

## Introduction

The cost-effectiveness threshold (CET) is undoubtedly a challenging subject that holds worldwide significance, as it directly relates to the economic value placed on a person’s life. In an ideal world with unlimited resources, it would be possible to finance all health-related interventions that improve health outcomes. However, we live in a world where human, financial, and natural resources are limited, even for the wealthiest countries. As resources are limited, we need to make difficult choices from the available options. When viewed as a tool to aid in the selection of cost-effective alternatives from the available options, the CET becomes a reasonable consideration [1].

The CET represents a monetary value that indicates the maximum acceptable amount to be paid for achieving a unit of health gain, often aggregated in quality-adjusted life years (QALYs) or disability-adjusted life years (DALYs). Through cost-effectiveness analysis (CEA), we can calculate the incremental cost-effectiveness ratio (ICER), which is then compared against the CET to determine whether an intervention offers a good value for money [2]. If the ICER falls at or below the threshold, the intervention is considered cost-effective [2].

The derivation of the CET value can be approached in various ways. One approach is to infer its value from past resource allocation decisions, e.g., the cost of treating end-stage renal disease [3]. Another approach involves capturing the willingness of a society to pay for an additional life year or a QALY through individual decisions or using the value of a statistical life (VSL) [4, 5]. An additional approach called “exhausting a fixed budget” can be implemented, in which a league table ranks health interventions by their ICERs; interventions with the lowest ICERs are financed first, followed by less cost-effective interventions until the budget is depleted [6]. One more approach that is widely adopted is to set the CET at 1–3 times the GDP per capita of a country [7]. The CET should ideally reflect society’s monetary valuation of health gains or the opportunity cost associated with disinvestment to adopt new technology [8].

Some countries opt for multiple thresholds due to varying priorities across diseases or patient groups [6]. These multiple-threshold systems may differentiate thresholds based on disease severity or rarity [9, 10].

In Egypt, the primary objective of implementing health technology assessment (HTA) is to enable evidence-based decision-making regarding the reimbursement of medical interventions [11]. However, without a formal CET in place, it becomes challenging to determine whether assessed interventions offer good value for money and merit reimbursement.

The need for a CET in Egypt has been highlighted in several studies. A study in 2013 by Elsisi et al. emphasized the importance of benchmarking the ICER derived from economic evaluations in Egypt [12]. More recently, Fasseeh et al. outlined an action plan endorsed by Egyptian decision-makers for the implementation of HTA, recommending the establishment of explicit multiple thresholds within a timeframe of 1–2 years [13]. Furthermore, the Egyptian Association for Health Economics (EAHE) encouraged the development of a local CET. The EAHE, as a national scientific association, brings together key stakeholders involved in HTA implementation in Egypt. Its mission is to raise awareness about the role of HTA in decision-making in Egypt and to provide policy recommendations based on scientific research and consensus among multiple stakeholders.

Our research was designed to address three key research questions: “How is the CET applied in different countries?”, “What is the correlation between the CET and the economic status of countries?”, and “What is the optimal CET framework for Egypt given the economic status of the country and the health care system?”.

Our study aimed to identify CET values across various countries and offer an overview of the global trends of CETs. These global trends are characterized by the growing adoption of multiple CETs, a practice that is becoming increasingly common in numerous countries worldwide [6, 9]. These multiple CETs are often differentiated based on disease severity or rarity [6, 9]. Exploring such information is intended to guide the creation of a tailored CET framework for Egypt, taking into account the local structure of its healthcare system.

## Methods

The development of the CET for Egypt was carried out in three distinct stages. First, we searched the literature to get insights on CETs, their application in healthcare, and their correlation with the economic status of countries. Our search also included a review of countries that report multiple CETs to identify prevailing global trends of the CET. Second, an expert panel was assembled to review the literature findings, engage in in-depth discussions, and formulate potential recommendations for the development of the CET framework. Finally, a workshop was conducted to validate and refine the draft CET framework and reach a consensus on the precise CET values that would best align with Egypt’s healthcare system.

### Cost-effectiveness threshold literature search

#### Search strategy

First, to identify countries with a CET, we used the World Health Organization (WHO) “list of HTA agencies worldwide” — as a starting point. This comprehensive list comprises *113* HTA agencies and networks across *63* countries. Since the list was last updated in 2014 [14], we augmented it using information from the websites of the European Network for Health Technology Assessment (EUnetHTA) and the International Network of Agencies for Health Technology Assessment (INAHTA) [15, 16]. Through this process, two additional countries were included in our analysis.

To gather CET values and details for each country, we followed a structured hierarchical approach. Firstly, we conducted thorough searches on the websites of the identified HTA agencies for each country. In cases where the HTA agency websites yielded insufficient data, we then referred to the respective Ministries of Health websites. To streamline this process, Google search engine was used *(site: URL/search term)* to search within HTA agencies and Ministries of Health websites. The search terms used were: (“Cost Effectiveness” OR “Cost-Effectiveness”) AND “Threshold”. We excluded non-English language websites to ensure efficiency and minimize complexity within the search.

If the previously mentioned sources did not yield relevant information about the CET, alternatively, we searched for the country’s economic evaluation guidelines through the Professional Society for Health Economics and Outcomes Research’s (ISPOR) website [17]. Finally, if no relevant data were retrieved using these sources, we searched the literature for publications about the CET or economic evaluations referencing the benchmarked CET using Google Scholar search engine. The search was limited to the English language and included only studies published since 2010. The search terms used were as follows: ((“Country” AND “Cost-Effectiveness”) OR (“Cost-Effectiveness” AND “Threshold”)).

#### Data extraction

For each identified study, we extracted the following: “Value of the threshold”, “Basis of the Threshold”, “Range of the threshold”, “Year of threshold captured”, and the “Type of the threshold”. The data extraction sheet was designed in Microsoft Excel.

For each country, we categorized the CET based on the approach used to derive it. Accordingly, the CET was based on either the gross domestic product (GDP) per capita, average wage, and adapted NICE (National Institute for Health and Clinical Excellence) threshold or a threshold with an unspecified basis.

Thresholds were either reported as a single-point estimate or a range of values. If only a single value was reported, we considered it as a single-point estimate threshold. When the CET was reported as a range, we used the lower value of the range for further calculations. Occasionally, the upper value of the range was used for specific technologies that met certain criteria, while the lower value of the range was used for common technologies in non-priority disease areas [18].

In terms of the type of threshold, it was either reported as explicit or implicit. Explicit thresholds were those officially published and available in the public domain, such as on an official website, while implicit thresholds were defined as those used for decision-making without formal publication. However, some countries did not have any defined thresholds; in these cases, the reported value in the study was inferred by the authors based on the context of a cost-effectiveness analysis conducted in that country. We have categorized these inferred values as “Benchmarked thresholds published in CEA”.

Most publications reported the threshold as cost per QALY; however, DALYs averted were used in some countries in the absence of QALY data. If multiple publications reported different threshold values for the same country, the latest publication was chosen to reflect the CET. If the date of threshold implementation was missing, we assumed it to be the publication date.

#### Data processing and analysis

For countries that linked their CET to the GDP per capita, we estimated the CET value by multiplying the 2019 GDP per capita values, which we obtained from the World Bank database [19], with the minimum multiplier reported. The 2019 GDP values for Cuba, Bhutan, and Taiwan were not present in the database, so we searched the knoema.com website for their values [20–22]. For countries that did not tie their CET to the GDP per capita, values reported in the country’s local currency were converted to United States dollars (USD) using the World Bank currency exchange rates for the year 2019 [23]. However, some publications did not report the values in their local currency, so we converted these values to the country’s local currency with the year’s exchange rate reported for that particular year and then converted them back to USD for the year 2019.

We used descriptive statistics to summarize the threshold’s source, range, basis, and value. To compare between different countries, each country’s CET value was divided by its GDP per capita. CET/GDP value was calculated and compared to the mean threshold for all included countries. To allow for subgroup analysis, countries were classified based on income groups and regions according to the World Bank classification [24].

### Multiple thresholds search

Exploring global trends of the CET relied on searching for countries implementing multiple or differential thresholds through Google Scholar and Google search engines. We used different combinations of keywords representing the domain “multiple thresholds” in the context of the health-related CET. The search term used was (“multiple” OR “differential” OR “end-of-life”) AND “Thresholds”.

We extracted relevant data including values of the multiple thresholds, country, year, and the basis for the multiple thresholds. The extracted data were then summarized and presented narratively.

### Egyptian cost-effectiveness threshold

Establishing a CET for Egypt should be based on the consensus of the various governmental bodies involved in national health care services. Accordingly, the process involved different stakeholders representing relevant governmental bodies in Egypt. Stakeholders were chosen based on convenient sampling methods and included those who had experience and knowledge about HTA and CEAs. The recommendation for the national CET in Egypt was developed through a stepwise approach. First (as previously stated), a literature search was conducted and used as guidance for developing the CET. Second, an expert panel was convened to review the literature findings, engage in discussions about potential recommendations for CET development, and create a draft CET framework. Finally, a workshop was conducted to validate the draft CET framework and reach a consensus on the precise CET values.

#### National experts’ panel

A panel consisting of 15 stakeholders representing various governmental organizations — Universal Procurement Authority (UPA), Egyptian Drug Authority (EDA), and Universal Health Insurance Authority (UHIA) — convened at the National Training Institute in Cairo, Egypt, on the 22nd of January 2021. The panel started by presenting the findings from the literature search concerning global CETs and multiple thresholds. This was followed by a guided focus group discussion, led by an international expert, on the applicability of these global practices at the national level.

During the discussion, a consensus was reached among the panelists that the CET should be used as a tool for negotiating reimbursement and pricing decisions rather than an inflexible rule. Furthermore, the panel emphasized the need to develop a national framework tailored to the structure of Egypt’s healthcare system, rather than adopting another country’s framework. Based on these discussions, four main recommendations were formulated as the initial steps toward the development of the CET framework.

Subsequently, two focus group meetings were held in the form of workshops, involving the same group of experts, to translate the stated recommendations into a draft framework for the CET in Egypt. A final workshop was then conducted to validate the CET framework and vote on the exact values to be included.

#### Workshop (voting)

The workshop included 19 experts representing relevant governmental sectors — UPA, UHIA, EDA, and the WHO — a list of experts, affiliations, and roles are presented in the supplement (Table S1). During this workshop, a survey was conducted where live voting was carried out anonymously through mobile phones using Mentimeter® software. The survey conducted during the workshop involved voting on several key parameters. Key parameters included the minimum and maximum GDP per capita multiplier, the minimum prevalence to consider a disease as rare or ultra-rare, the minimum and maximum threshold multiplier for rare diseases, and the multiplier for private insurance. Voting options are presented in Table 1. The median values of the voting results were used to eliminate the effect of outliers.

**Table 1:** Voting options

Questions	Options
What is the minimum GDP per capita multiplier?	0.5, 1.0, 1.5, 2.0
What is the maximum GDP per capita multiplier?	2.0, 2.5, 3.0, 3.5, 4.0, 4.5, 5.0
What is the minimum to consider something a rare disease?	1/1000, 1/2000, 1/3000, 1/4000, 1/5000
What prevalence is considered for an ultra-rare disease?	1/10,000, 1/20,000, 1/30,000, 1/40,000, 1/50,000, 1/60,000, 1/70,000, 1/80,000, 1/90,000, 1/100,000
What is the minimum rare disease multiplier?	1.0, 1.5, 2.0
What is the maximum rare disease multiplier?	2.0, 2.5, 3.0, 3.5, 4.0
What is the fixed multiplier for out-of-pocket or private insurance?	1.5, 2.0, 2.5, 3.0
Do you want to have a pilot?	Yes, no
When do you think it is feasible to revisit the recommendations?	6 months, 1 year, 2 years, 3 years

## Results

### Cost-effectiveness threshold literature search

Out of 65 countries initially identified, 7 were excluded from the analysis. Germany, Turkey, Croatia, and New Zealand reported the absence of an official CET, and no published values were available for these countries [25–28]. The CET values for Latvia, Uruguay, and Luxembourg were not available. Consequently, 58 countries were included in the final analysis. The included countries, along with their respective CET values and the basis of their threshold, are presented in Table 2.

**Table 2:** CET in USD (2019) and reported currency for all included countries

	**Country**	**Reported CET**	**Year of CET**^**b**^	**CET in USD 2019**	**Basis of the threshold**	**Reference**
**Explicit threshold**	Chile	18,586–55,758 USD	2016	14,896	GDP	[29]
	Czechia	1,200,000 CZK	2018	52,328	Not defined	[30]
	Hungary^a^		2017	49,427	GDP	[31]
	Ireland	20,000–45,000 EUR	2014	22,389	Not defined	HIQA [32]
	Netherlands	10,000–80,000 EUR	2015	11,195	Not defined	zorginstituutnederland [33]
	Poland	150,000 PLN	2016	46,786	GDP	[34]
	Portugal	10,000–100,000 EUR	2019	11,195	Not defined	INFRAMED [35]
	Slovakia	33,390–39,114 EUR	2018	37,379	Salary	[36]
	Thailand	160,000 THB	2015	9370	GDP	[37]
	UK	20,000–30,000 GBP	2019	25,528	NICE threshold	NICE [38]
**Implicit threshold**	Australia	50,000 AUD	2019	34,758	Not defined	[39]
	Belgium	33,000 EUR	2021	36,943	Not defined	[40]
	Brazil	8649–25,949 USD	2015	8717	GDP	[41]
	Canada	50,000 CAD	2017	37,685	Not defined	CADTH [42]
	Israel	50,000 ILS	2018	14,027	Not defined	[43]
	Italy	25,000–40,000 EU	2018	27,987	Not defined	[44]
	Norway	500,000 NOK	2014	56,818	Not defined	[45]
	Philippines	120,000 PHP	2019	3485	GDP	[46]
	South Korea	32,038,000 KRW	2016	31,762	GDP	[47]
	Spain	30,000 EUR	2019	33,584	Not defined	[48]
	Sweden	770,000–1,200,000 SEK	2017	81,410	Not defined	[49]
	Tunisia	11,086 USD	2018	9953	GDP	[50]
	USA	50,000–150,000 USD	2020	50,000	Not defined	ICER [51]
	Japan	5,000,000 JPY	2019	45,867	Not defined	[52]
**Benchmarked threshold in published cost-effectiveness analysis**	Argentina	24,1430 ARS	2017	10,006	GDP	[53]
	Austria	30,000–40,000 EUR	2016	33,584	Not defined	[54]
	Bhutan	2708 USD	2017	3357	GDP	[55]
	Bolivia	1758–5274 USD	2011	3552	GDP	[56]
	Bulgaria	39,619 BGN	2019	29,213	GDP	[57]
	China	23,050 USD	2014	30,785	GDP	[58]
	Colombia	15,795 USD	2015	19,297	GDP	[59]
	Costa Rica	6629–19,888 USD	2014	12,238	GDP	[60]
	Cuba	5702–17,106 USD	2015	9100	GDP	[61]
	Cyprus	20,517–60,000 EUR	2012	27,858	GDP	[62]
	Denmark	300,000 DKK	2013	44,981	Not defined	[63]
	Ecuador	6302–18,906 USD	2015	6184	GDP	[64]
	Estonia	52,390 EUR	2019	70,980	GDP	[65]
	Finland	40,000 EUR	2013	44,779	NICE threshold	[63]
	France	32,000 EUR	2015	40,494	GDP	[66]
	Ghana	1480 USD	2015	2202	GDP	[67]
	Greece	49,000 EUR	2019	58,748	GDP	[68]
	India	90,688–272,064 INR	2014	2104	GDP	[69]
	Indonesia	3475–10,425 USD	2013	4136	GDP	[70]
	Kazakhstan	37,805 USD	2015	29,193	GDP	[71]
	Malaysia	10,456–31,370 USD	2013	11,415	GDP	[72]
	Mexico	10,307–30,921 USD	2016	9863	GDP	[73]
	Paraguay	2516–7549 USD	2009	5415	GDP	[74]
	Peru	20,000–60,000 PEN	2017	6978	GDP	[75]
	Romania^a^		2020	12,920	GDP	[76]
	Russia	2,235,201.6 RUB	2020	34,755	GDP	[77]
	Slovenia	20,000 EUR	2018	25,739	GDP	[78]
	South Africa	38,500 ZAR	2015	3181	GDP	[79]
	Switzerland	30,000–50,000 CHF	2017	30,188	Not defined	[80]
	Taiwan	727,818 TWD	2016	25,873	GDP	[81]
	UAE	140,000 AED	2016	43,103	GDP	[82]
	Ukraine	11,700 USD	2012	10,977	GDP	[83]
	Vietnam^a^		2014	2715	GDP	[84]
	Lithuania	50,000–100,000 USD	2010	42,221	Not defined	[85]

^a^Actual value of CET itself was not reported. However, it was mentioned that GDP per capita is the base of the threshold, so CET was calculated accordingly

^b^Year of CET is the year where the threshold was reported. In the absence of a reported year, the date of publication of the study was assumed to be the year of the reported threshold

#### Data source, range, and basis of the threshold

The majority of the CET values, approximately 88%, were retrieved from publications about the economic evaluations referencing the benchmarked CET or publications discussing the CET directly. Only 10% of the CET values were reported on the websites of HTA agencies, while a mere 2% were obtained from the ISPOR guidelines.

Concerning the basis of the threshold, 64% of the countries tied their CET to the GDP per capita. Slovakia was the only country that linked its CET to a multiple of the average wage [36]. Aside from the UK, sometimes publications reference the adapted NICE threshold in countries with no defined CET [63], while 33% did not clearly define their basis. In terms of the threshold structure, more than half of the countries (55%) used a single threshold, while the remaining 45% employed a threshold range.

#### Cost-effectiveness threshold

##### International

**Fig. 1:**
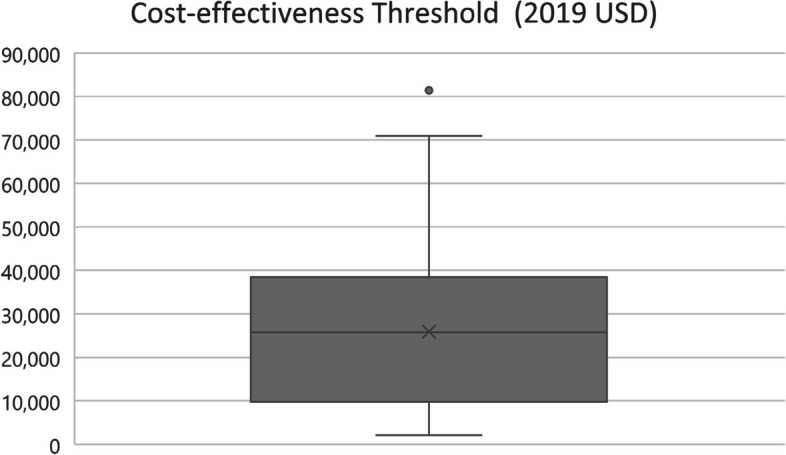
CET absolute value USD 2019 box-and-whisker diagram. CET, cost-effectiveness threshold. GDP, gross domestic product

**Fig. 2:**
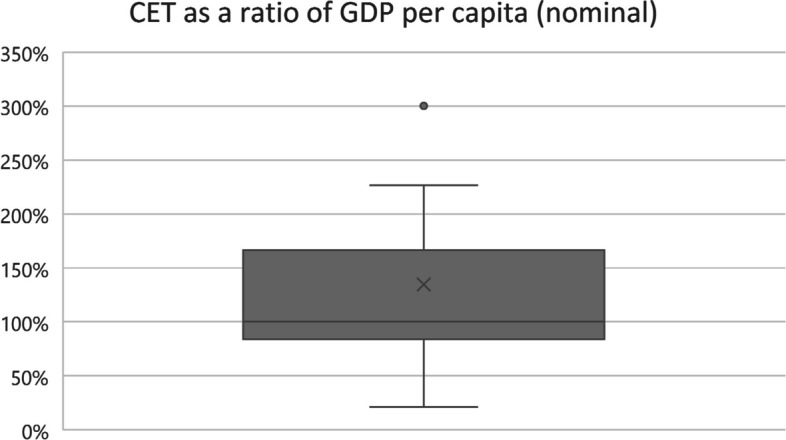
CET as a ratio of GDP per capita (nominal) box-and-whisker diagram. CET, cost-effectiveness threshold. GDP, gross domestic product

The absolute value of the lower CET varied across the included countries, ranging from 2000 to 81,000 USD (2019), with the lowest CET value recorded in India and the highest in Sweden [49, 69]. The mean and median absolute value of the CET across all countries was almost 26,000 USD (Fig. 1), while the average CET/GDP per capita was 135% (Fig. 2). Thus, on average, countries use a slightly higher value than their GDP per capita as the CET when considering the lower end of the threshold range.

We analyzed the thresholds based on geographical regions. The mean absolute CET values and the CET as a percentage of the GDP per capita differentiated by geographical regions are presented in the supplement (Tables S2, S3).

High-income countries were the most represented in the study (55%), followed by middle-income countries (29% upper-middle and 16% lower-middle), with no low-income country represented. Almost half of the countries (48%) were in Europe and Central Asia, whereas 19% were in Latin America and the Caribbean, 17% in East Asia &and the Pacific, and 5% in the Middle East and North Africa. The remaining regions (South Asia, sub-Saharan Africa, and North America) represented 3%, by two studies each.

#### Cost-effectiveness threshold as an absolute value

For each income group, the mean value of the CET increases proportionally with the income level. The mean CET absolute value for all included countries was calculated and referred to as “unstratified mean” to be further used in the comparison. For lower-middle-income countries, the threshold ranged from 8 to 42% of the unstratified mean, while the percentage of the mean CET to the unstratified mean was 18%. The threshold of the upper-middle-income countries had a broader range of 12–134% compared to the unstratified mean, with an average of 56%. On the other hand, the CET values of the high-income countries had a much wider range: 43–314% of the unstratified mean, with the highest average threshold of 146% (Table 3).

**Table 3:** CET average absolute value and CET as a percentage of GDP per capita per income group

**Income group average CET**	**Mean CET (USD 2019)**	**Mean/unstratified mean**	
Lower-middle income	4720	18%	
Upper-middle income	14,625	56%	
High income	37,829	146%	
**Income group CET/GDP per capita**	**Minimum**	**Mean**	**Maximum**
Lower-middle income	100%	144%	300%
Upper-middle income	53%	157%	300%
High income	21%	120%	300%

*CET* Cost-effectiveness threshold, *GDP* Gross domestic product

#### Cost-effectiveness threshold as a percentage of gross domestic product per capita

To facilitate comparison across countries regardless of their income level, we calculated the CET (lower CET in case of CET range) as a ratio of the GDP per capita. The minimum ratio observed in lower-middle-income countries was 100%, while high-income countries had a minimum ratio of 21%. All countries with a CET of less than 50% compared to their GDP per capita were high-income countries. Lower-middle and upper-middle-income countries had a relatively higher average CET/GDP (144% and 157%, respectively) compared to high-income countries (120%) (Table 3).

**Fig. 3:**
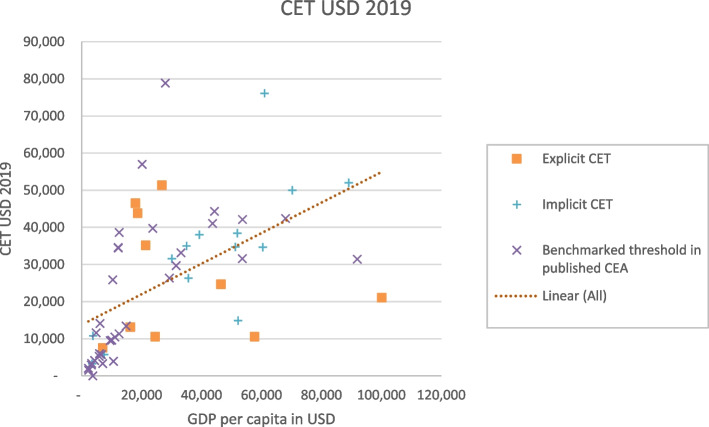
CET VS GDP per capita for types of thresholds using CET lower values. CET, cost-effectiveness threshold. GDP, gross domestic product

**Fig. 4:**
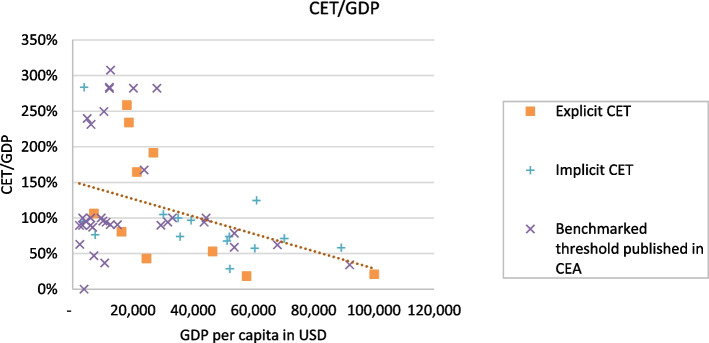
CET/GDP per capita vs GDP per capita for types of thresholds using CET lower values. CET, cost-effectiveness threshold. GDP, gross domestic product

The absolute value of the CET tends to increase with a country’s growing GDP per capita. However, when considering CET as a percentage of GDP per capita, it exhibits a regressive pattern. This means that countries with higher GDP per capita seem to have higher absolute values for the threshold, but the threshold constitutes a lower percentage of the country’s GDP per capita. Figures 3 and 4 illustrate the relationship between the absolute values of the CET, CET/GDP, and GDP per capita. The figures also represent types of threshold values stratified as explicit, implicit, and benchmarked thresholds published in CEA literature.

We analyzed the thresholds based on geographical regions. The mean absolute CET values and the CET as a percentage of the GDP per capita differentiated by geographical regions are presented in the supplement (Tables S2, S3).

### Multiple threshold search results

Based on the literature findings, some countries adopted different ICER thresholds for specific indications to accommodate the variability in costs and ethical concerns across disease states. The severity and rarity of the disease were identified as key factors for adopting higher thresholds. Additionally, several healthcare systems implemented multiple threshold systems to prioritize specific disease areas, such as oncology. Furthermore, one of the countries suggested a dual-payer system where a higher threshold could be applied to the private reimbursement system relative to the public one [86]. However, no evidence of implementing such a system was available [86].

For thresholds based on the severity of the disease, the Netherlands applied a range of 20,000, 50,000, and 80,000 EUR per QALY based on proportional shortfall estimates [9]. Furthermore, Norway’s proposed threshold was based on estimates of absolute shortfall (AS) and was divided into 6 severity classes ranging from NOK 275,000/QALY up to NOK 825,000/QALY [87]. Although the Norwegian government did not formally adopt the suggested AS classes and thresholds, they were informally used to inform healthcare allocation decisions [12].

Thresholds were also differentiated based on priority disease areas, such as oncology. France, for instance, uses a threshold up to 6 times its standard threshold for some oncology drugs (300,000 EUR/QALY) [10]. In Italy, a threshold of 87,330 EUR was reported to be used for the assessment of oncology products representing almost 3 times its standard threshold of 30,000 EUR [44, 88]. Canada adopts one and a half times the commonly used threshold of 50,000 CAD/QALY for oncology drugs ranging from 70,000 to 80,000 CAD /QALY [6]. The United States employs a broad range of thresholds between 150,000 and 300,000 USD per QALY, representing around 2–6 times the commonly used threshold of 50,000 to 150,000 USD/QALY [89]. In the UK, technologies providing “life-extending treatment at the end of life” are assessed according to a threshold of 50,000 GBP/QALY compared to the standard threshold of 20,000–30,000 GBP/QALY [90].

For rare diseases and orphan drugs, countries have established separate thresholds for assessment. For example, in Thailand, drugs that treat rare conditions are not assessed according to the conventional threshold of one time the GDP per capita per QALY [6]. The same occurs in France, where a higher threshold is used to assess drugs for rare conditions [10]. Furthermore, the Institute for Clinical and Economic Review (ICER) in the United States has discussed a range of up to 500,000 USD/QALY for ultra-rare diseases (10 times the standard threshold). Likewise, NICE adopted a variable threshold of up to 300,000 GBP per QALY depending on the magnitude of the health gains [91].

In Japan, a slightly different approach is taken for implementing multiple thresholds. A threshold range of 7.5–15 million Japanese yen is applied for products with special considerations representing one and a half times the standard threshold [52]. Products with special consideration categorization apply to rare disease indications, pediatric labeling indications, or cancer therapies [12].

In South Africa, there are two parallel insurance systems: private and public. Culyer suggested that in the short term, the cost-effectiveness threshold of public insurance should be set at a lower value than the private one [86].

Sweden applies a four-level threshold approach based on the societal value of the intervention: interventions delivering a very high societal value are assessed according to a threshold of more than 1,000,000 SKR/QALY or life years; high societal value — 1,000,000–500,000 SKR; middle societal value — 500,000–100,000 SKR; and low societal value — less than 100,000 SKR [6]. In Australia, an implicit threshold is used based on clinical need and the availability of alternatives. Interventions with an ICER value of less than 45,000 AUD were usually recommended for reimbursement, while those with a higher ICER were only recommended for reimbursement if they provided evidence of high clinical need or the absence of alternatives [2, 92].

### Egyptian cost-effectiveness threshold

#### National expert panel

Based on the literature findings, in which most countries use GDP per capita as a basis for defining their threshold, the panelists proposed linking the national CET to the economic status of Egypt, represented by the GDP per capita. Furthermore, they suggested a preliminary threshold between one and a half to three times the GDP per capita, specified for each specific health intervention based on its QALY gain. The notion of setting the threshold value in relation to the QALY gain of the intervention was used to mirror the severity of the disease. Since QALY is a metric that considers both the quantity and quality of life produced by healthcare interventions, the severity of a condition inversely affects the quality of life. Consequently, a higher QALY gain reflects a substantial improvement in health status, especially for more severe conditions. However, the proposed range of the threshold (1.5–3 times) was subjected to change based on the voting sessions conducted afterward. Experts also recommended having a differential threshold for the public (public reimbursement) and the private (out-of-pocket payment) settings. To foster equity, rare diseases were proposed to have a higher threshold that varies based on the rarity of the disease.

Finally, panelists highlighted that the CET should only be employed for judging the value of innovative, single-source technologies. Single-source technologies are defined as technologies that do not have any alternatives with the same active ingredient (generics, biosimilars). For these technologies, economic evaluations should be conducted to compare their ICER against the CET. In contrast, interventions having more than one alternative (e.g., generics and biosimilars) will not require cost-effectiveness analysis and will not be assessed using the CET. They also recommended using multicriteria decision analysis (MCDA) for multisource drugs to determine the best option among alternatives. The panel discussed the importance of disease burden studies in accurately depicting the real burden of each disease; by understanding the real impact of the diseases, resources can be allocated based on scientific evidence and prioritized accordingly. The main recommendations concluded by the national expert panel are presented in Table 4.

**Table 4:** Expert panel recommendations

**Expert Panel Recommendations**
Linking the CET to the GDP per capita
A different threshold for rare diseases that varies by rarity
A higher threshold for out-of-pocket/private insurance compared to public reimbursement
The threshold should be based on QALY gain where it can vary from 1.5 to 3.0 times the GDP per capita.

*CET* Cost-effectiveness threshold, *GDP* Gross domestic product

Two focus group meetings in the form of workshops were then held, engaging the same experts from the expert panel discussion. The workshops aimed to develop a CET framework by translating the four main recommendations from the expert panel discussion into a draft framework that could be voted upon. The foundation of the framework is to propose a public CET.

Initially, the panelists suggested using the absolute shortfall to reflect the severity of the disease, similar to other countries like Norway [87]. However, subsequent discussions with experts in the local market pointed out challenges in calculating the absolute shortfall (AS) due to the scarcity of local data. As a result, the experts recommended using the incremental relative QALY gain (IRQG) approach [93].

The proposed public threshold structure is based on the severity of the disease and incorporates four threshold values corresponding to different IRQG ranges. Health technologies that fall within a higher IRQG range will have a higher threshold, as shown in Table 5. IRQG can be calculated using the following equation:

$$IRQG=\frac{{QALY}_{new \,technology}-{QALY}_{comparator}}{{QALY}_{new \,technology}}$$

**Table 5:** CET for public reimbursement with a multiplier for out-of-pocket pricing

Incremental relative QALYs gain (IRQG)	Cost-effectiveness threshold (CET)	Public (public reimbursement)	Private (out-of-pocket payment) 2 × multiplier
		CET value based on GDP^a^ 2022 in **EGP**	CET value based on GDP^a^ 2022 in **EGP**
0.00–0.10	1.0 × GDP per capita	82,294	164,588
0.10–0.25	2.0 × GDP per capita	164,588	329,176
0.25–0.50	2.5 × GDP per capita	205,735	411,470
0.50–1.00	3.0 × GDP per capita	246,882	493,765

*CET* Cost-effectiveness threshold, *IRQG* Incremental relative QALYs gain, *GDP* Gross domestic product

^a^GDP values are derived from World Bank data

Experts agreed to use multipliers applied on top of the base CET represented in Table 5 to calculate the threshold for the private sector and a further multiplier for rare diseases. To reduce complexity in the CET framework, experts opted for a fixed multiplier to calculate the private (out-of-pocket payment) threshold based on the public threshold structure. For rare diseases, different multipliers will be used based on the rarity of the target disease.

#### Workshop (voting)

Experts voted for a threshold of one-three times the GDP per capita for the minimum and maximum multiplier of the public threshold. Furthermore, they recommended a two-times multiplier for the private threshold on top of the IRQG multiplier (Table 5).

In terms of severity multipliers, the experts agreed that if the value of the IRQG is between 0.00 and 0.10, the threshold value should be one times the latest GDP per capita published by governmental agencies or available in the World Bank database expressed in EGP. If the IRQG is between 0.10 and 0.25, the threshold is going to be two times the GDP per capita; while in the case of the IRQG is between 0.25 and 0.5, the threshold will be two and a half times the GDP per capita; and finally, if the IRQG is between 0.5 and 1.0, the threshold should be three times the GDP per capita (Table 5).

In terms of the rarity of the disease, an exponential scale rounded to the nearest thousand was developed to assess rarity. Based on the participants’ votes, the minimum prevalence to consider a disease rare was 1/4000 (one person developing the disease in 4000 people), and for a disease to be regarded as ultra-rare, it was 1/90,000. According to the outcome of the participants’ votes, a minimum rarity multiplier of one and a half times for a prevalence of 1/4000 was adopted, while the maximum multiplier was three times for ultra-rare diseases. The rarity multiplier featured a scale starting from one and a half to three times the GDP per capita. Multiplier values related to rarity are described in Table 6.

**Table 6:** Rare disease factor

Rarity	Multiplicator
1/4000–1/5999	1.5
1/6000–1/7999	1.8
1/8000–1/17,999	2.1
1/16,000–1/35,999	2.4
1/36,000–1/89,999	2.7
≥ 1/90,000	3

Although participants voted on defining the multipliers for rare diseases, they had a significant disagreement regarding the inclusion of rarity as the sole criterion in determining the differential threshold of rare diseases due to the absence of local prevalence data, and the importance of other factors such as the availability of existing treatment, or budget impact.

Finally, participants recommended a pilot phase for 1 year to test the framework in the real world and evaluate if any modification will be required.

## Discussion

CETs are necessary for decision-makers to judge whether extra benefits offered by new interventions are worth the additional cost [2, 94]. Since Egypt started implementing HTA for reimbursement of health technologies, there is an urgent need to establish CETs to support objective and transparent decision-making [11].

Our review of the literature revealed that the majority of countries tend to tie their CET to the GDP per capita. According to Leech et al. [95], 66% of CEAs published in LMICs between 2000 and 2015 used GDP-based CETs. Furthermore, Kazibawe et al. [96] examined CEA studies in LMICs that were published between 2015 and 2020 where their results emphasized that GDP-based CETs remained the most frequently used in CEA studies (84.3%). The aforementioned studies relied on published CETs in CEA, while in our study, to ensure reliability, a hierarchical approach was followed to identify CETs in different countries where official HTA agencies websites were searched first followed by Ministries of Health, ISPOR database, and lastly CEA.

Several studies explored the conceptualization of CETs and the methodologies for estimating CETs, with some estimating these thresholds across multiple countries based on opportunity costs [4, 97]. Our study shares similarities with the research conducted by Schwarz et al. [6], as both aimed to identify and characterize CETs in multiple countries, examining whether they were explicitly stated or implicit and general or specific to particular indications. However, our study differs from Schwarz’s research in several key areas. While Schwarz’s study focused on 10 countries, our study covered a wider range, examining the CET in over 58 countries. This broader scope enabled us to provide a more comprehensive global perspective on the CET [6]. Methodologically, there are also significant differences. Schwarz et al. employed a systematic review and expert surveys within their respective countries [6]. In contrast, our study heavily relied on literature searches as the main method for data collection. We followed a hierarchical approach to prioritize reliability. This approach was better suited for our study due to the large number of countries included. Although incorporating expert surveys across all countries could have potentially enhanced the precision of our values, it would have posed considerable challenges given the extensive scale of our research.

Numerous studies have been conducted with the objective of establishing national CETs for their respective countries. For example, Kovács et al. aimed to set a new CET for Hungary by reviewing the CETs in 26 European countries [93]. Similar to our study, they used the IRQG to reflect disease severity due to the lack of sufficient information for using the absolute shortfall. Hungary set a threshold of 1.5 times the GDP per capita, which could increase up to 3 times depending on the IRQG. For interventions used in rare diseases, the threshold could rise to 10 times the GDP per capita depending on QALY gain. In contrast, our study broadened the scope to cover 58 countries worldwide, rather than focusing solely on Europe. We employed a threshold of 1–3 times the GDP per capita, also based on the IRQG to reflect disease severity. For interventions used in rare diseases, we applied a multiplier ranging from 1.5 to 3 times depending on the rarity of the disease. Both studies related their threshold to the GDP per capita as it serves as a good proxy indicator for a country’s changing health budget.

Another study by Al-Jedai et al., conducted in Saudi Arabia, developed their threshold by estimating the marginal cost per unit of health produced by the healthcare system [98]. They used the income elasticity of the value of health to arrive at a threshold of 50,000 to 75,000 SAR, depending on opportunity costs. In contrast, this approach requires comprehensive local data about health spending, health outcomes, and other factors [98]. This data was not available, or reliable at the time of conducting our study, and at the same time, it is not transferable across different countries, regions, or sectors. For instance, at the time of the study, the most recent National Health Accounts was more than 10 years old and was published for the year 2008/2009 [99]. Therefore, national experts decided to use the GDP per capita as it is the more feasible approach for setting a national CET. Moreover, referencing the GDP per capita reduces the need for frequent updates of the CET as it reflects changes in the economy. Furthermore, Jedai et al. highlighted that there are other factors of interest beyond population health, where the value of health benefits can vary depending on the context as mentioned in the article “health benefits for patients suffering rare or severe diseases may be given greater weight than health benefits for the average member of the general population”. This recommendation was fulfilled in our study where we considered multiple thresholds for disease severity and rarity.

In our study, the expert panel recommended using the GDP approach for establishing CETs since it reflects the country’s economic status. Based on global data, absolute CET was positively correlated with the countries’ GDP per capita; however, the ratio CET/GDP per capita showed a negative correlation with the countries’ GDP per capita. Most of the data found in the literature concerning CET values were not explicitly published in HTA agencies or Ministry of Health websites. The exclusion of non-English publications might explain this, or transparency may be still an issue even in countries that have fully implemented HTA systems. Nevertheless, HTA should not be used as an inflexible rule but rather as a tool for negotiating reimbursement and pricing decisions. Using CETs as a tool provides an opportunity to increase the number of interventions that can be reimbursed, ultimately contributing to improved patient access. This approach enables decision-makers to compare different options based on predefined values and criteria [96]. Conforming with that, Egyptian experts recommended using an implicit threshold which will be piloted for 1 year to ensure it aligns with the desired outcomes of the healthcare system.

Having the same threshold for all technologies ignores equity, particularly in the case of rare diseases, and leads to a decrease in patient access to expensive yet necessary health technologies [2]. Thus, multiple thresholds are implemented in several jurisdictions based on the local environment as they can facilitate reimbursement of public priority drugs. Furthermore, multiple thresholds give a fair opportunity for drugs that treat rare diseases to be reimbursed, taking into account the relative social value of QALYs in different population groups. The expert panel agreed to use higher CET according to the disease rarity, the incremental gain of the intervention, and the healthcare sector. Finally, private sector thresholds were recommended to be set at double the value of the public sector threshold. This policy, proposed by Culyer in South Africa [86], aligns with the fact that out-of-pocket prices tend to be higher; these higher prices serve as exposed prices for reference by other countries. Using multiple (differential) CETs enhances the responsiveness of the healthcare financing system, as it supports equity and considers societal values in healthcare decision-making when assessing rare diseases.

The adoption of CETs in Egypt promotes transparent decision-making [100]. Furthermore, in case of low-middle-income countries, implementing an explicit threshold is expected to lower the public reimbursement price of innovative pharmaceuticals. This price reduction is facilitated by the implementation of managed entry agreements (MEAs), and not by direct discounts to block international price referencing from higher-income countries [101].

It should be noted that the threshold values were captured at the time of this study and are subject to change over time. New thresholds have been introduced by some countries, such as the Kingdom of Saudi Arabia (KSA), and a recent reference for Slovenia has been acknowledged by our co-authors [98, 102]. However, it was not feasible to incorporate the new values into our analysis, so we included them in the supplement for reference.

### Limitations

The majority of CET values were obtained from economic evaluation publications rather than official HTA agency or Ministry of Health websites, as these sources often did not publish data in English. This approach may have resulted in the omission of some published or explicit thresholds from our research. Also, the countries included in the study were mainly wealthier ones, with no low-income countries represented, primarily because these countries are currently not implementing HTA and do not have defined CET values. Another limitation included the absence of patient organizations’ involvement in the development of the CET. Although we recognize the importance of incorporating patient perspectives into health policy decisions, patient organizations in Egypt are not yet well-established, and their inclusion may have introduced potential variability and uncertainty into our results.

## Conclusion

CET values are typically linked to the GDP per capita. Several countries use multiple thresholds for interventions based on rarity, severity, and type of disease or other factors. Egyptian experts decided to use the CET value of one to three times the GDP per capita value. In addition, they considered the possibility of using multiple thresholds based on incremental relative QALY gain and the rarity of the disease.

## Supplementary Information

Supplementary Material 1: Table S1. Experts affiliations and roles. Table S2. CET average absolute value per geographical area. Table S3. CET as a percentage of GDP per capita per geographical area.

## Data Availability

The datasets supporting the conclusions of this article are available from the corresponding author upon reasonable request.

## Abbreviations

AS: Absolute shortfall
CEA: Cost-effectiveness analysis
CET: Cost-effectiveness threshold
DALY: Disability-adjusted life years
EDA: Egyptian Drug Authority
EUnetHTA: European Network for Health Technology Assessment
GDP: Gross domestic product
HTA: Health technology assessment
ICER: Incremental cost-effectiveness ratio
ICER: Institute for Clinical and Economic Review
INAHTA: International Network of Agencies for Health Technology Assessment
IRQG: Incremental relative QALY gain
ISPOR: Professional Society for Health Economics and Outcomes Research
MCDA: Multi-criteria decision analysis
MEA: Managed entry agreements
NICE: National Institute for Health and Clinical Excellence
QALY: Quality-adjusted life year
UHIA: Universal Health Insurance Authority
UPA: Universal Procurement Authority
VSL: Value of a statistical life
WHO: World Health Organization
