# Correction: Framework for developing cost-effectiveness analysis threshold: the case of Egypt

**DOI:** 10.1186/s42506-024-00178-4

**Published:** 2024-12-04

**Authors:** Ahmad N. Fasseeh, Nada Korra, Baher Elezbawy, Amal S. Sedrak, Mary Gamal, Randa Eldessouki, Mariam Eldebeiky, Mohsen George, Ahmed Seyam, Asmaa Abourawash, Ahmed Y. Khalifa, Mayada Shaheen, Sherif Abaza, Zoltán Kaló

**Affiliations:** 1 Faculty of Pharmacy, Alexandria University, Alexandria, Egypt; 2 Syreon Middle East, Alexandria, Egypt; 3 Department of Public Health, Cairo University, Cairo, Egypt; 4 Egyptian Authority for Unifed Procurement, Medical Supply and Technology Management, Cairo, Egypt; 5 Department of Community Health, Fayoum University, Fayoum, Egypt; 6 Universal Health Insurance Authority, Cairo, Egypt; 7 Egyptian Drug Authority, Cairo, Egypt; 8 World Health Organization Representative Office, Cairo, Egypt; 9 Roche, Cairo, Egypt; 10 Center for Health Technology Assessment, Semmelweis University, Budapest, Hungary; 11 Syreon Research Institute, Budapest, Hungary

**Correction: J Egypt Public Health Assoc 99, 12 (2024)**

**https://doi.org/10.1186/s42506-024-00159-7**

Following publication of the original article [1], we have been notified that the body text of the article contained incorrectly published information.

Table 2 contained a typographical mistake.

It was:

Implicit threshold / Italy—25,000–45,000 EU

It was updated to:

Implicit threshold / Italy—25,000–40,000 EU

Also, one sentence on page 9, paragraph 3 was published incorrectly.

It was:

In Italy, a threshold of 87,330 EUR was reported to be used for the assessment of oncology products representing almost 3 times its standard threshold of 30,000 EUR [88].

It was updated to:

In Italy, a threshold of 87,330 EUR was reported to be used for the assessment of oncology products representing almost 3 times its standard threshold of 30,000 EUR [44, 88].

The original article was updated.
