# Supplementary material for: Framework for developing cost-effectiveness analysis threshold: the case of Egypt
Source: J Egypt Public Health Assoc. 2024 Jun 3;99:12. doi: 10.1186/s42506-024-00159-7 (PMC11144683; doi:10.1186/s42506-024-00159-7)
Supplement: Supplementary file 1 — Supplementary Material 1: Table S1. Experts affiliations and roles. Table S2. CET average absolute value per geographical area. Table S3. CET as a percentage of GDP per capita per geographical area. [file 42506_2024_159_MOESM1_ESM.pdf]

Framework for Developing Cost-Effectiveness Analysis Threshold: The Case of Egypt:

Ahmad Nader Fasseeh[1,2] (PhD), Nada Korra[1] (PharmD), Baher Elezbawy[1] (MPH), Amal Samir Sedrak[3,4] (PhD), Mary Gamal[4] (DBA), Randa Eldessouki[5] (PhD), Mariam Eldebeiky[4] (MBA), Mohsen George[6] (PhD), Ahmed Seyam[6](MSc), Asmaa Abourawash[7] (BSc), Ahmed Yehia Khalifa[8] (MSc), Mayada Shaheen[9] (MBA), Sherif Abaza[10] (MBA), Zoltán Kaló[11,12] (PhD)

1. *Syreon Middle East, Alexandria, Egypt*
2. *Eötvös Loránd University, Budapest, Hungary*
3. *Associate Professor Public Health, Cairo University, Cairo, Egypt*
4. *Egyptian Authority for Unified Procurement, Medical Supply and Technology Management, Cairo, Egypt*
5. *Fayoum university, Fayoum, Egypt*
6. *Universal Health Insurance Authority, Cairo, Egypt*
7. *Egyptian Drug Authority, Cairo, Egypt*
8. *World Health Organization Representative Office, Cairo, Egypt*
9. *Roche, Cairo, Egypt*
10. *Syreon Middle East, Cairo, Egypt*
11. *Center for Health Technology Assessment, Semmelweis University, Budapest, Hungary*
12. *Syreon Research Institute, Budapest, Hungary*

Corresponding Author:

Nada Korra

Address: 142 Elshaheed Galal Desouky street, Alexandria

Country: Egypt

Email: nada.korra@syreon.eu

ORCHID details: 0000-0003-0611-3789

Experts involved in the final workshop

Table S1 Experts affiliations and roles

Experts	Affiliation	Roles
Expert 1	Egyptian Authority for Unified Procurement, Medical Supply and Technology Management, Cairo, Egypt	Technology reassessment department manager
Expert 2	Egyptian Authority for Unified Procurement, Medical Supply and Technology Management, Cairo, Egypt	Pharmacist at health technology assessment (HTA) unit
Expert 3	Egyptian Authority for Unified Procurement, Medical Supply and Technology Management, Cairo, Egypt	Head of Technology Management
Expert 4	Egyptian Authority for Unified Procurement, Medical Supply and Technology Management, Cairo, Egypt	Pharmacist
Expert 5	Egyptian Drug Authority, Cairo, Egypt	Pharmaco-economist
Expert 6	Universal Health Insurance Authority, Cairo, Egypt	Technical advisor to UHIA
Expert 7	Egyptian Authority for Unified Procurement, Medical Supply and Technology Management, Cairo, Egypt	Pharmacist
Expert 8	1. Associate Professor Public Health, Cairo University, Cairo, Egypt<br>2. Egyptian Authority for Unified Procurement, Medical Supply and Technology Management, Cairo, Egypt	1. Associate Professor of public health<br>2. Head of general administration of training and community awareness at UPA
Expert 9	Egyptian Drug Authority, Cairo, Egypt	Pharmacist in Pharmacoeconomics unit
Expert 10	Roche Egypt	Health Policy Lead
Expert 11	Egyptian Authority for Unified Procurement, Medical Supply and Technology Management, Cairo, Egypt	Pharmacist
Expert 12	UPA	Pharmacist
Expert 13	EDA	Pharmaco-economist
Expert 14	Universal Health Insurance Authority, Cairo, Egypt	Director of HTA, Health Economics, and Health Systems Research
Expert 15	WHO Representative Office in Egypt	Health Economist
Expert 16	Egyptian Drug Authority, Cairo, Egypt	Head of pharmaco-economics unit
Expert 17	Egyptian Authority for Unified Procurement, Medical Supply and Technology Management, Cairo, Egypt	General manager of Needs collection and assessment department
Expert 18	Egyptian Authority for Unified Procurement, Medical Supply and Technology Management, Cairo, Egypt	Pharmacist
Expert 19	Egyptian Drug Authority, Cairo, Egypt	Health Economist

Cost-effectiveness threshold analysis of geographical regions

Based on the geographic regions, North America had the highest mean threshold (169%) compared to the unstratified mean of all countries. At the same time, Sub-Saharan Africa and South Asia had the lowest mean of 10% and 11% respectively of the unstratified mean. In Europe & Central Asia, the mean value of the threshold was 142% compared to the unstratified mean. The Middle East & North Africa region averaged 86% compared to all countries as an absolute value of CET. The mean CET values are presented in the following table:

Table S2: CET average absolute value per geographical area

Region	Mean CET (USD 2019)	Mean/Unstratified mean
South Asia	2,731	11%
Europe & Central Asia	36,771	142%
The Middle East & North Africa	22,361	86%
East Asia & Pacific	20,017	77%
Sub-Saharan Africa	2,691	10%
Latin America & Caribbean	9,659	37%
North America	43,842	169%
All regions	25,806	100%

CET: cost-effectiveness threshold

There was a considerable variation in the Middle East & North Africa region's CET/GDP per capita value, ranging from 32%-300%. In Latin America & the Caribbean, the range was 100%-300%. In Europe and Central Asia, the CET/GDP per capita ranges from 21%-300%. The CET as a percentage of the GDP is presented in supplementary table 2 (Table S2).

Table S3: CET as a percentage of GDP per capita per geographical area

Region CET/GDP per capita	Minimum	Mean	Maximum
South Asia	100%	100%	100%
Europe & Central Asia	21%	156%	300%
The Middle East & North Africa	32%	144%	300%
East Asia & Pacific	63%	120%	300%
Sub-Saharan Africa	53%	77%	100%
Latin America & Caribbean	100%	118%	300%
North America	77%	79%	82%
All regions	21%	135%	300%

CET: cost-effectiveness threshold, GDP: gross domestic product

New and Recent Threshold Values

Country	CET	Year	Reference
Kingdom of Saudi Arabia (KSA)	50,000-70,000 SAR	2023	(1)
Slovenia	25,000 EUR	2022	(2)

1. Al-Jedai AH, Lomas J, Almudaiheem HY, Al-Ruthia YSH, Alghamdi S, Awad N, et al. Informing a cost-effectiveness threshold for Saudi Arabia. Journal of Medical Economics. 2023;26(1):128-38.
2. Burgess C, Kujawski S, Lapornik A, Bencina G, Pawaskar M. The Long-Term Clinical and Economic Impact of Universal Varicella Vaccination in Slovenia. Journal of Health Economics and Outcomes Research. 2022;9(2):95.
